# Evaluation of electron Monte Carlo algorithm accuracy for dose calculations in extended source‐to‐surface distances electron beam therapy

**DOI:** 10.1002/acm2.70237

**Published:** 2025-08-29

**Authors:** Naohito Ono, Makoto Omiya, Manami Sugiyama, Masaki Oshima

**Affiliations:** ^1^ Department of Radiological Technology Juntendo University Shizuoka Hospital Izunokuni Shizuoka Japan; ^2^ Department of Radiation Oncology Juntendo University, Graduate School of Medicine Bunkyo‐ku Tokyo Japan

**Keywords:** electron beam therapy, extended source‐to‐surface distance, Monte Carlo simulation, radiation treatment planning system

## Abstract

**Background:**

Extending the source‐to‐surface distance (SSD) is an effective approach to cover a large irradiation area in electron beam therapy for large planning target volumes (PTVs). However, the accuracy of dose calculations at extended SSDs has not been fully validated.

**Purpose:**

This study evaluated the dose calculation accuracy of the electron Monte Carlo (eMC) algorithm implemented in Varian's Eclipse radiation therapy planning system (RTPS) under extended SSD conditions.

**Methods:**

Simulations were conducted using Eclipse version 13.6 for 6, 12, and 18 MeV electron beams, with SSDs ranging from 100 to 140 cm. A 25 cm × 25 cm applicator and a virtual water phantom were utilized to compute percent depth dose (PDD), off‐axis ratio (OAR), and output factor (OPF). The calculated values were compared with measured data and independent Monte Carlo (MC) simulations performed using Particle and Heavy Ion Transport System (PHITS), referred to as PHITS‐MC in this study.

**Results:**

The eMC algorithm achieved high accuracy along the central axis, with PDD deviations within 2 mm and OPF differences within 3% across all SSDs, including 140 cm. However, eMC exhibited increasing deviations in OAR field size (>3 mm) at SSD ≥ 120 cm. A detailed parameter‐based analysis further revealed underperformance in OAR calculations at field peripheries for low‐energy beams (6 MeV), compared with PHITS‐MC.

**Conclusion:**

The findings delineate the performance and limitations of the eMC algorithm under extended SSD conditions. These limitations should be considered in algorithm evaluation and quality assurance processes. The results provide guidance for algorithm assessment and may serve as a foundation for future studies to explore its clinical relevance in large‐field electron beam therapy.

## 1 INTRODUCTION

Electron beam therapy using a linear accelerator (LINAC) is typically performed with single portal irradiation from the superficial direction, as it targets superficial tumors. The treatable depth is determined by the 90% depth (commonly denoted as R_90_, hereafter referred to as D_90_) or 80% depth (D_80_) on the percent depth dose (PDD) curve, which can be easily estimated based on the electron beam energy used.^1^, ^2^ Additionally, since the transverse radiation field shape is defined by the applicators and cutouts, treatment planning can be done without a radiation therapy planning system (RTPS) for a visible planning target volume (PTV). As a result, the use of RTPS in electron beam therapy is less common than in x‐ray therapy, which involves multidirectional treatment using a multi‐leaf collimator. Historically, pencil beam algorithms were the primary method for electron beam dose calculations in commercial RTPSs^3^; however, their limited accuracy—particularly in small fields and heterogeneous media—has been well documented,^4^, ^5^ which contributed to the limited clinical use of RTPSs for electron beam therapy.

However, in clinical practice, the shape, size, and composition of the target PTVs vary significantly, making electron beam therapy planning challenging in certain cases. One such case is the treatment of small irradiation fields or heterogeneous media where lateral scatter equilibrium (LSE) of electrons is not maintained. Changes in the shape of the PDD and off‐axis ratio (OAR) make it difficult to predict the treatable range^6^; thus, RTPS visual simulations are desirable. Many studies have examined the accuracy of the dose calculation algorithm for such treatments. Recently, Monte Carlo (MC) dose calculation algorithms have become available for RTPS, with improved accuracy reported in small irradiation fields and heterogeneous media.^3^, ^7^

Another challenging planning situation in electron beam therapy is the treatment of large PTVs. In particular, postoperative radiation therapy for keloids, which proliferates beyond the original wound margins, often requires large irradiation fields.^8^ Low‐energy electron beams, such as 6 MeV, are predominantly used for extensive keloids located on anatomical sites, such as the anterior chest or back, and treatment typically begins within 24 h postoperatively.^9^ Assuming the homogeneity of the target volume is often feasible in relatively flat anatomical regions, such as the chest wall, and thus a practical approach is required to cover large PTVs using a single irradiation field. The feasibility of treatment simulation using an RTPS provides a valuable basis for algorithm evaluation and quality assurance in large‐field electron beam therapy.

Electron beam irradiation is typically performed using a standardized applicator attached to the machine. For the TrueBeam linear accelerator by Varian Medical Systems, Palo Alto, California, USA (hereafter referred to as Varian), the maximum applicator size is 25 cm × 25 cm. For larger irradiation fields, the field must either be divided or the source‐to‐surface distance (SSD) must be extended. The field division method requires consideration of junctions between fields, which can complicate treatment planning and actual irradiation. Conversely, the SSD extension method is simpler and avoids interference with the applicator, making it a more common choice. However, extending the SSD can result in energy reduction, changes in output factor (OPF), increased penumbra, and decreased dose uniformity within the irradiation field.^10^ Consequently, simple predictions are difficult, and RTPS simulations are desirable. However, the accuracy of dose calculations when using extended SSDs is not guaranteed, and the American Association of Physicists in Medicine (AAPM) Task Group (TG)‐70 has emphasized the need for preverification.^10^ Conducting preverification enables efficient and safe treatment using RTPSs.

The accuracy of the algorithm is determined by the beam model and registered data. Eclipse version 13.6, a Varian RTPS, incorporates the electron Monte Carlo (eMC) algorithm for electron beam therapy calculations. The algorithm comprises two main components: an electron beam source model and a transport model.^11^ The electron beam source model simulates the accelerated electrons, along with electrons and photons generated in the LINAC head structures. It includes five sub‐sources: main source, jaw source, scraper source, edge electrons, and transmitted photons, each with specific properties to improve computational efficiency. The transport model calculates particle transport in the patient's body, using the macro Monte Carlo (macro MC) method to determine dose delivery by primary and secondary particles along their trajectory. To ensure agreement with measured PDDs and OARs, a library of precomputed probability density functions for local geometries is used to simplify particle transport and reduce computation time. This study investigates the accuracy of dose calculations when eMC is used for extended SSD. Because eMC is based on beam data measured at the facility's own LINAC, a comparison with a general‐purpose MC code, independent of the actual measurements, was also performed.

Although the feasibility of extended SSD electron beam therapy has been previously recognized in selected clinical contexts, such as treatment of superficial tumors requiring large irradiation fields, only limited work has been done to characterize algorithmic performance under these conditions.^12^, ^13^, ^14^, ^15^, ^16^ In particular, there is a lack of systematic investigation into SSD‐dependent changes in geometric parameters—such as PDD, OAR, and OPF—and how these may vary with beam energy. Such factors are crucial for ensuring safe and accurate treatment delivery when extended SSDs are used in practice, particularly beyond 120 cm. A more detailed assessment of these aspects is necessary to validate the robustness of dose calculation algorithms like eMC under extended clinical setups. In this study, we systematically investigated these geometric parameters using both eMC and Particle and Heavy Ion Transport System (PHITS) simulations under multiple energy conditions and extended SSDs up to 140 cm. Rather than simply avoiding applicator interference, this study primarily aimed to provide detailed and systematic information on algorithm performance and limitations, based on homogeneous phantom‐based evaluation, to support dose calculation evaluation and quality assurance in large‐field electron beam therapy planning, particularly for cases such as postoperative keloid treatment.

## 2 METHODS

### 2.1 Simulation with eMC

Electron beam therapy was simulated using eMC with the Varian RTPS, Eclipse version 13.6. The dose distribution during electron beam irradiation was modeled. For beam modeling in eMC, the required data included the PDD and OAR measured at the maximum irradiation field, consisting of the upper and lower jaws without an applicator, the PDD measured with an applicator, and the absolute dose measured at the maximum dose depth (D_max_) on the central axis. These measurements were performed following the manual provided by Varian during the LINAC installation^17^ and RTPS commissioning, in accordance with AAPM TG–53 guidelines.^18^

In this study, a virtual water phantom with dimensions of 50 cm × 50 cm × 20 cm (lateral × longitudinal × vertical) was initially created using the contouring mode in Eclipse. Given that the SSD expansion led to an irradiation field size of 25 cm × 25 cm or larger, the lateral and longitudinal directions were set to 50 cm, while the vertical direction was set to 20 cm, reflecting the assumed patient body thickness. The phantom was positioned at an SSD of 100 cm and electron beam therapy was simulated. This setup served as the reference condition, and the SSD was adjusted to 105, 110, 120, 130, and 140 cm to calculate the extended SSD dose. Three different electron beam energies (6, 12, and 18 MeV) were used to evaluate the variation in calculation accuracy based on energy.

Variable parameters for dose calculation in eMC include statistical uncertainty, calculation grid size, number of particle histories, dose threshold for uncertainty, smoothing method, and smoothing level. Optimal values for these parameters have been reported in several studies. ^5^, ^19^, ^20^, ^21^ In this research, based on literature findings and computation time considerations for clinical practice, the statistical uncertainty was set to 2%, the grid size to 1 mm for 6 MeV, and 2 mm for 12 and 18 MeV. The smoothing method and level were set to 3D Gaussian medium. However, dose prescription normalization was not applied due to the influence of these parameters, and the dose distribution was simulated using 200 MU irradiation.

To compare the calculated dose distributions, the PDD was plotted in the vertical direction along the central axis from the phantom surface, and the OAR was plotted in the lateral direction at D_max_ for each irradiation condition using the RTPS dose profile function. The PDDs and OARs were extracted as text files and analyzed using spreadsheet software. For comparison with the measured values and PHITS‐MC data, the dose distributions calculated by eMC were resampled at 1 mm intervals for PDD and 2 mm intervals for OAR. The PDD and OAR were normalized such that the D_max_ and central axis (CAX) of the PDD and OAR were set to 100%.

The depths (mm) at which the output reached 90%, 80%, 50%, 20%, and 10% when D_max_ was 100% were calculated as D_90_, D_80_, D_50_, D_20_, and D_10_, respectively, as evaluation indices for the PDD. D_100_ was excluded from the evaluation because small fluctuations in the measured values can cause significant changes, particularly at high energies.^20^ The OAR evaluation indices were L_90%_, L_80%_, L_50%_, L_20%_, R _90%_, R_80%_, R_50%_, and R_20%_ for the left and right positions, where the CAX output reached 90%, 80%, 50%, and 20%. The irradiation field size, penumbra, L_80%_–R_80%_ distance, and L_90%_–R_90%_ distance were also calculated. The irradiation field size was determined by the distance between the L_50%_ and R_50%_ points, while the penumbra was calculated as the average distance between the L_80%_ and L_20%_ points and the R_80%_ and R_20%_ points.

To assess the accuracy of the output at extended SSD when using eMC, the D_max_ dose was obtained from the treatment plan for 200 MU irradiation under each measurement condition. These values were normalized for each energy value, using the output of the reference condition (SSD 100 cm) as 100%, and the OPF (%) was calculated.

### 2.2 MC simulations with PHITS

The PHITS version 3.24 was used for MC simulations, independent of the measured data. PHITS is a general‐purpose MC code (PHITS‐MC) that models various radiation behaviors in any material using nuclear reaction models and nuclear data. It can simulate particle transport in electron therapy by defining the LINAC structure and source.^22^ The PDD and OAR calculations were performed using PHITS under the same irradiation conditions as described in the Section 2.1. The PHITS simulations and the measurements of Section 2.3 were conducted using the methodology employed in our previous study.^23^

**FIGURE 1:**
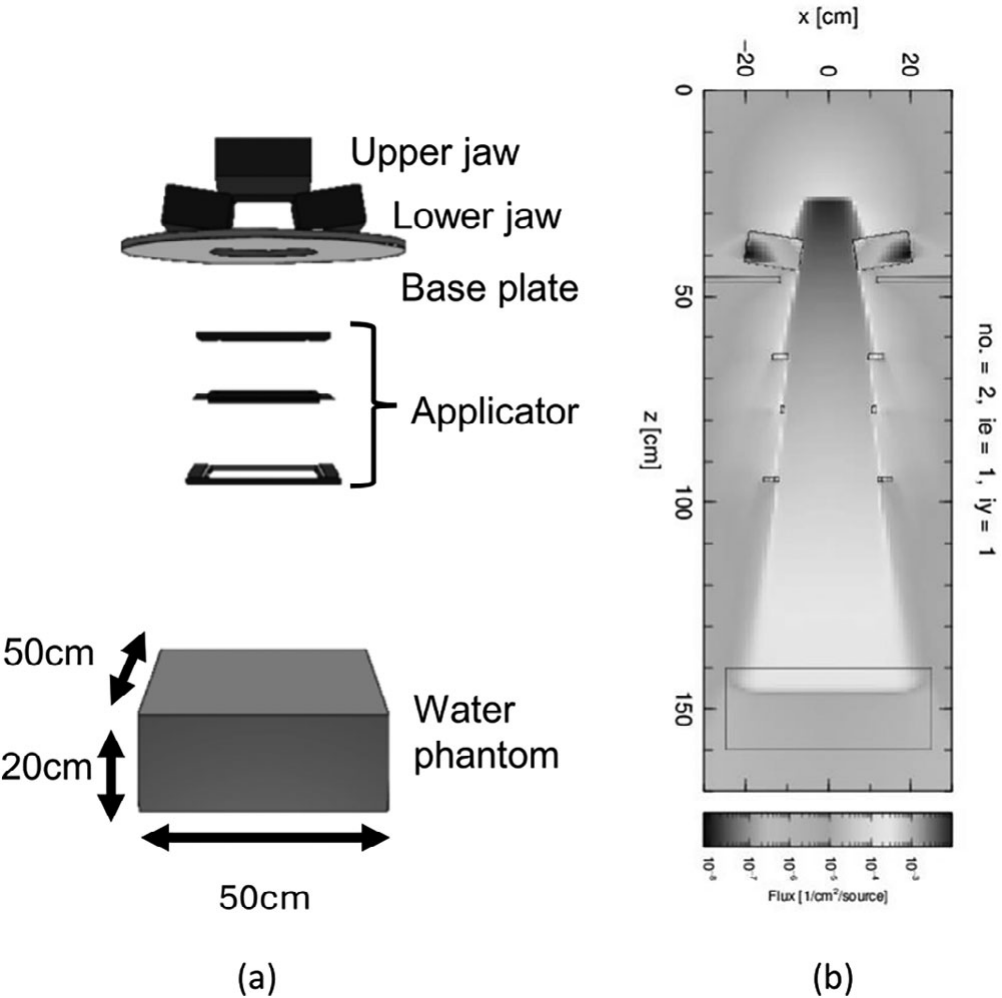
MC simulation of 12 MeV electron beam irradiation using PHITS (SSD 140 cm). (a) Modeling of the TrueBeam structure. (b) Fluence distribution of the electron beam.

First, a TrueBeam structure with a 25 cm × 25 cm applicator attached to the gantry was modeled as a preliminary step. The composition, electron density, and position were determined based on the materials provided by the vendor.^24^ Since the structure above the upper jaw of the TrueBeam has not been published, the phase space file provided by the vendor was used as the source.^25^ Figures 1a and b show the modeled structure and the corresponding electron fluence distribution at an SSD of 140 cm for the 12 MeV beam, respectively. The irradiation field size, formed by the upper and lower jaws of the TrueBeam, was defined based on the energy used and the applicator size. In this study, modeling was conducted to achieve 32, 30, and 27 cm for 6, 12, and 18 MeV energies with a 25 cm × 25 cm applicator. The electron beam applicator consists of a three‐stage aperture, with the top two stages made from zinc alloy (Al 8.4%, Cu 1%, MgZn 0.02%, remainder Zn), and the bottom stage made of CERROTRU (Bi 58%, Ti 42%). A virtual water phantom measuring 50 cm × 50 cm × 20 cm (lateral × longitudinal × vertical) was created at an SSD of 100 cm, and dose calculations were performed under both reference conditions and extended SSD. For electron and photon transport calculations, PHITS internally employs the Electron Gamma Shower version 5 algorithm, which was used in this study for dose calculations. The number of particle histories simulated was set to 4–6 × 10⁸ to ensure that the statistical uncertainty at D_max_ was less than 1% for all energies and SSDs. The grid size (lateral × longitudinal × vertical) for the calculations was 5 mm × 5 mm × 1 mm for the PDD to improve resolution in the vertical direction and 2 mm × 5 mm × 2 mm for the OAR. The calculated PDD and OAR were smoothed using the simple moving average method. The OPF was calculated as in the previous section, using the dose value of the voxel at D_max_ for each irradiation condition.

### 2.3 Measurement of the PDD and OAR

**FIGURE 2:**
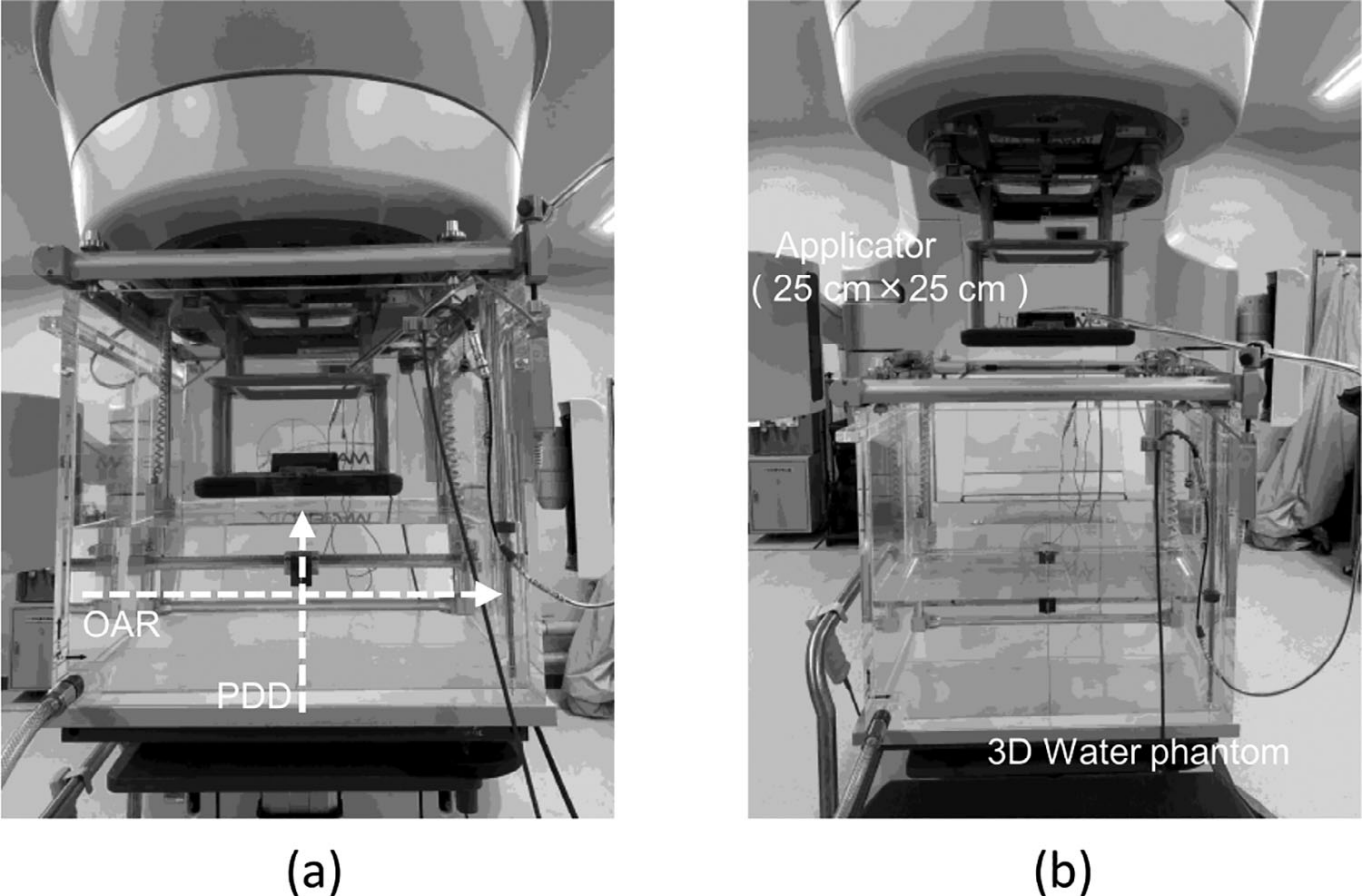
Configuration of the 3D water phantom during PDD and OAR measurements. (a) SSD 100 cm. (b) SSD 140 cm.

To replicate the geometrical in Section 2.1, an accessory mount and a 25 cm × 25 cm applicator were attached to the TrueBeam gantry. A three‐dimensional (3D) phantom, Blue Phantom2 (IBA Dosimetry, Schwarzenbruck, Germany), filled with water to a depth of 20 cm, was positioned where the SSD was 100 cm. For measurements with the extended SSD, the phantom was shifted in the vertical direction to set the SSD. Figure 2a illustrates the measurement setup at an SSD of 100 cm, while Figure 2b shows the setup with an extended SSD of 140 cm achieved by vertically shifting the water phantom. The dosimeter used was a silicon diode dosimeter 3G‐pSi EFD (IBA Dosimetry), featuring a high resolution of 1.6 mm in effective detector diameter and 0.08 mm in effective detector thickness. This dosimeter, which has a gradual change in the stopping power ratio, was used for direct measurement of the electron beam PDD and beam data collection in eMC modeling. An ionization chamber dosimeter CC13 (IBA Dosimetry), with a sensitive volume of 0.13 cm^3^, was placed at the inner edge of the irradiation field as a reference dosimeter to minimize output fluctuation during measurements. Measurements and result analysis were conducted using OmniPro‐Accept 7 (IBA Dosimetry).

PDD and OAR measurements were conducted under both reference and extended SSD conditions, as simulated in Section 2.1, using energies of 6, 12, and 18 MeV. The dose rates were set to 500 MU/min, with the measurement modes being step‐by‐step for PDD and continuous for OAR. PDD measurements were taken from the deep side to avoid surface tension effects, and OAR measurements were performed laterally at D_max_ for each energy value. Both measurements were taken at 1 mm intervals, with OAR resampled at 2 mm intervals for comparison with eMC and PHITS‐MC data. Additionally, PDD was normalized to D_max_, and OAR was normalized to 100% CAX using analysis software, with OAR also centered and symmetrized.

### 2.4 Output measurement at the extended SSD

To measure the output at the extended SSD, the Blue Phantom2 was positioned as described in Section 2.3. A NACP parallel‐plate ionization chamber (IBA Dosimetry) with a sensitive volume of 0.16 cm^3^ was used as the dosimeter, and a RAMTEC Duo (Toyo Medic) electrometer was employed. The dosimeter was placed at D_max_ in the water phantom for each irradiation condition, and 200 MU were delivered at a dose rate of 500 MU/min. Output measurements were taken for both the reference condition and extended SSD at energies of 6, 12, and 18 MeV. The OPF (%) was calculated from the measured output using the same procedure as in Section 2.1 and compared with the values calculated by PHITS‐MC and eMC.

### 2.5 Accuracy metric

Each PDD, OAR, and OPF parameter was assessed using a parameter‐based evaluation approach, following the methodology described by Hu et al.^20^ In line with the general acceptance criteria recommended in the AAPM Medical Physics Practice Guideline 5.a, ^26^ a 3 mm criterion was applied to geometric parameters (depth positions of D_90_, D_80_, D_50_, D_20_, D_10_ for PDD; field size and penumbra for OAR), and a 3% criterion was applied to dose differences (OPF and mean dose difference within the central 80% of the OAR profile). Gamma analysis and global pass‐rate evaluations were not performed in this study, to ensure a transparent and reproducible parameter‐based assessment under homogeneous water phantom conditions.

## 3 RESULTS

**FIGURE 3:**
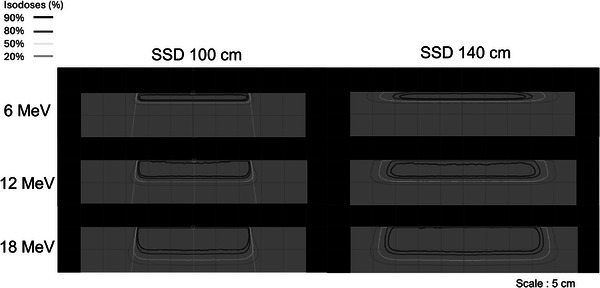
Dose distribution of electron beam irradiation simulated using eMC (SSD 100 and 140 cm). The isodose curves represent 90%, 80%, 50%, and 20% of the dose, normalized to 100% at D_max_ under each condition.

Figure 3 illustrates representative isodose distributions calculated using the eMC algorithm at SSDs of 100 and 140 cm. The 90%, 80%, 50%, and 20% isodose curves are normalized to the respective D_max_ values, visually exhibiting the expansion of the irradiation field and changes in dose gradients associated with increasing SSD. Figure 1 shows the geometry modeled in the PHITS‐MC simulation, whereas Figure 2 illustrates the experimental setup used to acquire measurement data under equivalent SSD conditions. These figures serve to confirm the geometric consistency between the simulation and measurement conditions. Altogether, these visualizations support the validity of the subsequent quantitative comparisons presented in the following sections.

### 3.1 Measured and simulated PDD and OAR under the reference conditions

**FIGURE 4:**
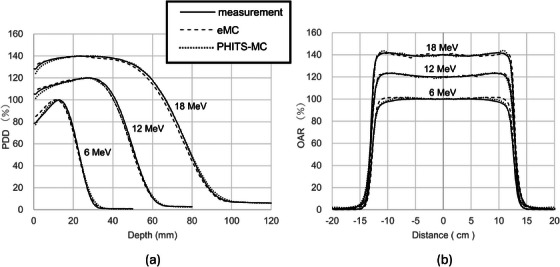
(a) PDD curves for 6, 12, and 18 MeV electron beams. Each curve is normalized to its respective D_max_. (b) OAR profiles assessed at D_max_ and normalized to the corresponding CAX dose value for each energy. To facilitate visual comparison of dose distributions across different energies, energy‐specific vertical offsets (e.g., 100%, 120%, 140%) were employed. This method is consistent with Rodrigues et al. (2016, Med Phys) and does not imply differences in absolute dose.

To improve visual clarity and facilitate comparison across different energies, the PDD and OAR curves in Figure 4 were normalized using energy‐specific scaling factors (e.g., 100%, 120%, 140%). PDD curves were normalized to the D_max_ and OAR curves to the CAX value. This convention was adopted to improve interpretability within a single figure and does not represent actual differences in absolute dose. A similar approach has been employed in previous studies, including Rodrigues et al.^25^ Figure 4a displays the measured and simulated PDDs at 100 cm SSD using a 25 cm × 25 cm applicator, alongside the eMC and PHITS‐MC simulations. Additionally, the D_90_, D_80_, D_50_, D_20_, and D_10_ values are presented in Table 1. The D_90_, D_80_, D_50_, D_20_, and D_10_ values for both eMC and PHITS‐MC matched the measured values within 2 mm, regardless of the energy.

**TABLE 1:** Measured and simulated PDD parameters under reference conditions.

Energy (MeV)	Depth	Measurement	eMC	*Difference*	PHITS‐MC	*Difference*
6	D_90_	17.2	16.3	*−0.9*	16.8	*−0.4*
	D_80_	19.1	18.4	*−0.7*	18.8	*−0.3*
	D_50_	23.1	22.7	*−0.4*	23.1	*0.0*
	D_20_	27.1	27.1	*0.1*	27.6	*0.5*
	D_10_	29.0	29.4	*0.4*	30.0	*1.0*
12	D_90_	38.8	37.6	*−1.2*	37.7	*−1.1*
	D_80_	42.4	41.3	*−1.1*	41.4	*−1.0*
	D_50_	49.5	48.7	*−0.8*	49.0	*−0.5*
	D_20_	56.3	55.9	*−0.4*	56.5	*0.2*
	D_10_	59.7	59.6	*−0.1*	60.3	*0.7*
18	D_90_	55.4	53.7	*−1.7*	55.6	*0.2*
	D_80_	62.4	61.0	*−1.4*	62.3	*−0.1*
	D_50_	74.7	73.6	*−1.1*	74.8	*0.1*
	D_20_	85.5	84.8	*−0.7*	86.2	*0.7*
	D_10_	91.4	90.7	*−0.7*	92.8	*1.4*

*Note*: All values are in mm.

The italic values in the tables are used to distinguish the “difference” date from the actual “measured values”.

Figure 4b shows the OARs from the actual measurements and the eMC and PHITS‐MC simulations at SSD 100 cm using a 25 cm × 25 cm applicator. Table 2 provides the penumbra, field size, L_80%_–R_80%_ distance, and L_90%_–R_90%_ distance. eMC and PHITS‐MC agreed with the measurements within 1.5 mm for the penumbra and within 1 mm for the field size, regardless of the energy.

**TABLE 2:** Measured and simulated OAR parameters under reference conditions.

Energy (MeV)	Distance	Measurement	eMC	*Difference*	PHITS‐MC	*Difference*
6	Penumbra	11.5	10.3	*−1.2*	12.5	*1.0*
	Field size	254.7	255.3	*0.5*	254.8	*0.0*
	L_80%_−R_80%_	243.1	245.2	*2.2*	242.1	*−1.0*
	L_90%_−R_90%_	234.3	239.7	*5.4*	233.7	*−0.5*
12	Penumbra	12.1	12.2	*0.1*	13.2	*1.1*
	Field size	259.9	260.3	*0.4*	260.0	*0.1*
	L_80%_−R_80%_	248.7	249.1	*0.4*	24.7.8	*−0.9*
	L_90%_−R_90%_	242.7	243.7	*1.0*	241.4	*−1.3*
18	Penumbra	8.2	8.1	*−0.1*	9.5	*1.2*
	Field size	258.9	258.4	*−0.5*	258.8	*−0.1*
	L_80%_−R_80%_	250.9	250.8	*−0.2*	249.7	*−1.2*
	L_90%_−R_90%_	246.4	246.9	*0.5*	245.1	*−1.3*

*Note*: All values are in mm.

The italic values in the tables are used to distinguish the “difference” date from the actual “measured values”.

For the L_80%_–R_80%_ and L_90%_–R_90%_ distances, eMC and PHITS‐MC matched the measured values within 1.5 mm at 12 and 18 MeV. However, at 6 MeV, the L_80%_–R_80%_ and L_90%_–R_90%_ distances were within 1.5 mm for PHITS‐MC, while for eMC, the L_80%_–R_80%_ distance was +2.2 mm, and the L_90%_–R_90%_ distance was +5.4 mm for eMC.

The parameter‐based evaluation at the reference SSD of 100 cm showed that eMC achieved agreement within 3 mm for all PDD depth indices (D_90_, D_80_, D_50_, D_20_, D_10_) and for all OAR geometric parameters, including field size, penumbra, and within 3% for mean dose difference within the central 80% of the field. PHITS‐MC demonstrated comparable accuracy for both PDD and OAR parameters at SSD 100 cm.

### 3.2 Measured and simulated PDD for the expanded SSD

**FIGURE 5:**
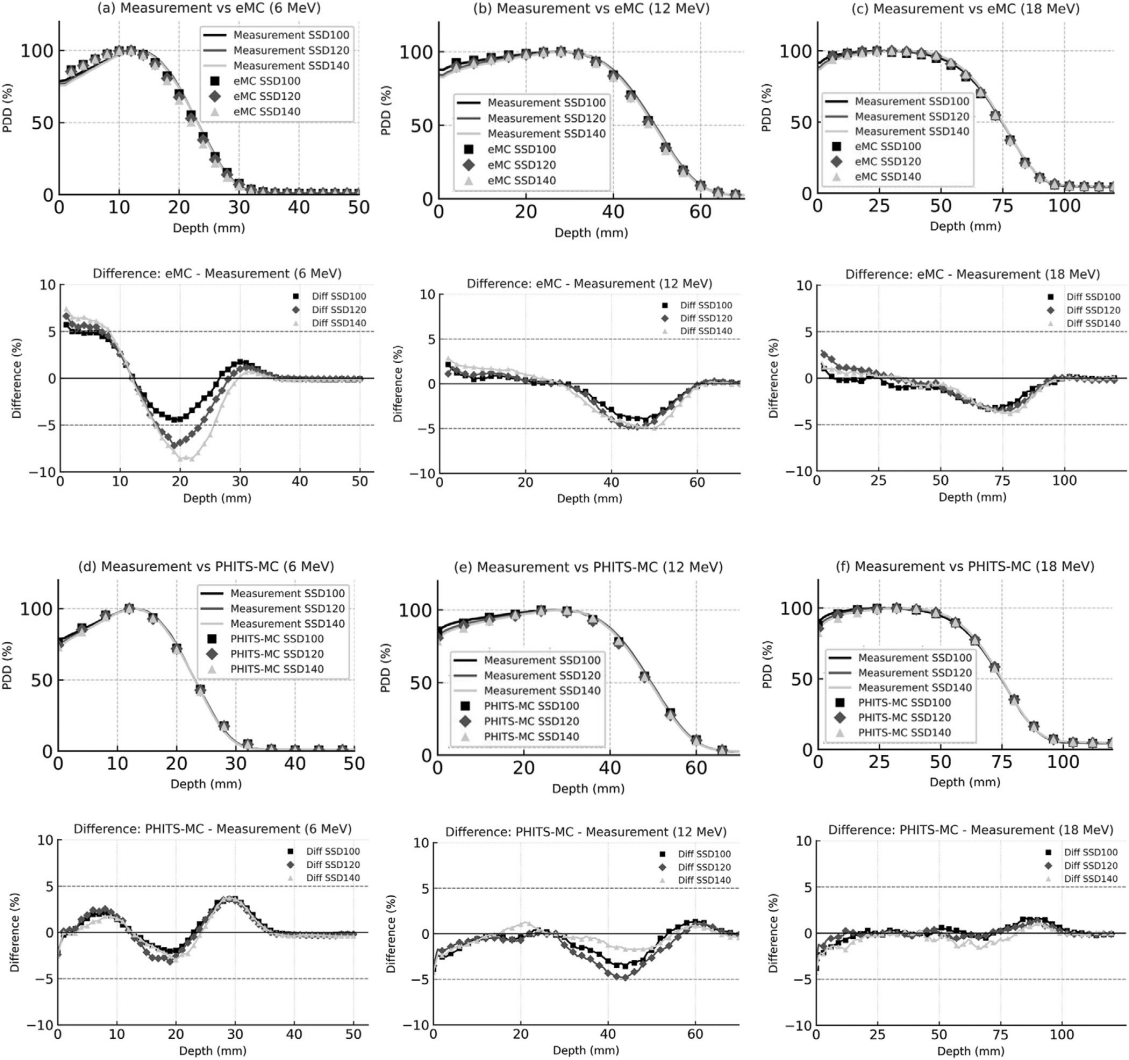
PDD profiles at SSDs of 100, 120, and 140 cm for 6, 12, and 18 MeV electron beams. Panels (a)–(c) compare measurement and eMC simulation results at 6, 12, and 18 MeV, respectively. Panels (d)–(f) show corresponding comparisons between measurement and PHITS‐MC simulation results. All profiles are normalized to their respective D_max_ to enable direct comparison. Solid lines indicate measured data, while simulation results are shown using markers. Square, diamond, and triangle markers represent SSDs of 100, 120, and 140 cm, respectively, for both eMC and PHITS‐MC. For clarity, energy‐specific subplots are used to prevent overplotting and ensure readability. To enhance interpretability, each profile is accompanied by a corresponding difference plot, where the measured values are subtracted from the simulated ones (i.e., eMC − Measurement or PHITS‐MC − Measurement), allowing visualization of SSD‐dependent discrepancies.

Figure 5 presents a comparative analysis of the PDD curves from measurements, eMC, and PHITS‐MC simulations at three representative SSDs—100, 120, and 140 cm—for 6, 12, and 18 MeV beams. To enhance visual clarity, each PDD profile is accompanied by a corresponding difference plot (eMC − Measurement or PHITS‐MC − Measurement) shown below the main graph. These SSDs were selected to reflect standard, moderately extended, and highly extended clinical setups. Table 3 shows the measured values of D_90_, D_80_, D_50_, D_20_, and D_10_, along with the differences (in mm) between the measured and calculated values from PHITS‐MC and eMC. The D_90_, D_80_, D_50_, D_20_, and D_10_ values from PHITS‐MC and eMC were within 2 mm of the measured values. However, the surface dose in the buildup region generally decreased with the SSD expansion for all energies (Figure 5). This trend was consistently observed in the measured values across the SSD changes, but some fluctuations were seen in the eMC and PHITS‐MC simulations, indicating variability.

**TABLE 3:** Differences (mm) between the measured and simulated PDD parameters for extended SSDs.

(a) D_90_ (mm)		SSD (cm)					
Energy (MeV)	D_90_ (mm)	100	105	110	120	130	140
6	Measurement	17.2	17.3	17.3	17.3	17.3	17.2
	*eMC difference*	*−0.9*	*−1.1*	*−0.9*	*−1.3*	*−1.4*	*−1.3*
	*PHITS‐MC difference*	*−0.4*	*−0.4*	*−0.5*	*−0.6*	*−0.6*	*−0.4*
12	Measurement	38.8	38.8	38.8	38.9	38.9	38.6
	*eMC difference*	*−1.2*	*−1.6*	*−1.5*	*−1.4*	*−1.7*	*−1.6*
	*PHITS‐MC difference*	*−1.1*	*−1.2*	*−1.1*	*−1.6*	*−1.2*	*−0.3*
18	Measurement	55.4	55.6	55.8	56.3	56.8	56.7
	*eMC difference*	*−1.7*	*−2.2*	*−1.7*	*−1.5*	*−1.9*	*−0.9*
	*PHITS‐MC difference*	*0.2*	*−0.5*	*−0.2*	*−0.6*	*0.0*	*−0.8*

(b) D_80_ (mm)		SSD (cm)					
Energy (MeV)	D_80_ (mm)	100	105	110	120	130	140
6	Measurement	19.1	19.1	19.1	19.2	19.1	19.0
	*eMC difference*	*−0.7*	*−0.9*	*−0.7*	*−1.1*	*−1.2*	*−1.2*
	*PHITS‐MC difference*	*−0.3*	*−0.3*	*−0.4*	*−0.5*	*−0.5*	*−0.4*
12	Measurement	42.4	42.4	42.4	42.5	42.4	42.1
	*eMC difference*	*−1.1*	*−1.5*	*−1.4*	*−1.4*	*−1.6*	*−1.3*
	*PHITS‐MC difference*	*−1.0*	*−0.9*	*−1.1*	*−1.4*	*−1.0*	*−0.4*
18	Measurement	62.4	62.5	62.6	62.9	63.2	63.1
	*eMC difference*	*−1.4*	*−1.5*	*−1.6*	*−1.4*	*−1.4*	*−1.2*
	*PHITS‐MC difference*	*−0.1*	*−0.2*	*−0.5*	*−0.1*	*−0.3*	*−0.4*

(c) D_50_ (mm)		SSD (cm)					
Energy (MeV)	D_50_ (mm)	100	105	110	120	130	140
6	Measurement	23.1	23.1	23.1	23.1	23.0	23.0
	*eMC difference*	*−0.4*	*−0.5*	*−0.4*	*−0.7*	*−0.8*	*−1.0*
	*PHITS‐MC difference*	*0.0*	*0.0*	*−0.1*	*0.0*	*−0.1*	*−0.2*
12	Measurement	49.5	49.5	49.5	49.5	49.4	49.2
	*eMC difference*	*−0.8*	*−1.1*	*−1.0*	*−1.0*	*−1.4*	*−1.1*
	*PHITS‐MC difference*	*−0.5*	*−0.5*	*−0.6*	*−0.6*	*−0.6*	*−0.3*
18	Measurement	74.7	74.7	74.8	74.9	74.9	74.8
	*eMC difference*	*−1.1*	*−1.1*	*−1.2*	*−1.2*	*−1.5*	*−1.1*
	*PHITS‐MC difference*	*0.1*	*−0.2*	*0.0*	*0.1*	*−0.2*	*−0.2*

(d) D_20_ (mm)		SSD (cm)					
Energy (MeV)	D_20_ (mm)	100	105	110	120	130	140
6	Measurement	27.1	27.1	27.1	27.0	27.0	27.0
	*eMC difference*	*0.1*	*0.0*	*0.0*	*−0.2*	*−0.4*	*−0.6*
	*PHITS‐MC difference*	*0.5*	*0.5*	*0.5*	*0.5*	*0.5*	*0.5*
12	Measurement	56.3	56.3	56.2	56.2	56.2	56.0
	*eMC difference*	*−0.4*	*−0.7*	*−0.6*	*−0.4*	*−0.8*	*−0.7*
	*PHITS‐MC difference*	*0.2*	*0.1*	*0.2*	*−0.1*	*−0.2*	*0.1*
18	Measurement	85.5	85.5	85.6	85.6	85.6	85.5
	*eMC difference*	*−0.7*	*−0.8*	*−0.4*	*−0.7*	*−0.9*	*−0.9*
	*PHITS‐MC difference*	*0.7*	*0.7*	*0.6*	*0.4*	*0.6*	*0.3*

(e) D_10_ (mm)		SSD (cm)					
Energy (MeV)	D_10_ (mm)	100	105	110	120	130	140
6	Measurement	29.0	29.0	29.0	28.9	28.9	28.9
	*eMC difference*	*0.4*	*0.3*	*0.4*	*0.1*	*0.0*	*−0.2*
	*PHITS‐MC difference*	*1.0*	*1.1*	*1.0*	*1.0*	*1.1*	*1.0*
12	Measurement	59.7	59.7	59.7	59.6	59.6	59.4
	*eMC difference*	*−0.1*	*−0.4*	*−0.3*	*−0.1*	*−0.4*	*−0.3*
	*PHITS‐MC difference*	*0.7*	*0.5*	*0.5*	*0.5*	*0.4*	*0.4*
18	Measurement	91.4	91.4	91.5	91.6	91.6	91.5
	*eMC difference*	*−0.7*	*−0.8*	*−0.4*	*−0.7*	*−0.9*	*−0.9*
	*PHITS‐MC difference*	*1.4*	*1.0*	*0.9*	*0.8*	*0.9*	*0.9*

*Note*: All values are in mm.

Difference (mm) = Simulation value − Measurement value.

The parameter‐based evaluation at extended SSDs (SSD > 100 cm) showed that eMC achieved agreement within 3 mm for all PDD depth indices across all energies. PHITS‐MC also maintained agreement within 3 mm for all PDD indices under the extended SSD conditions.

### 3.3 Measured and simulated OAR for extended SSD

**FIGURE 6:**
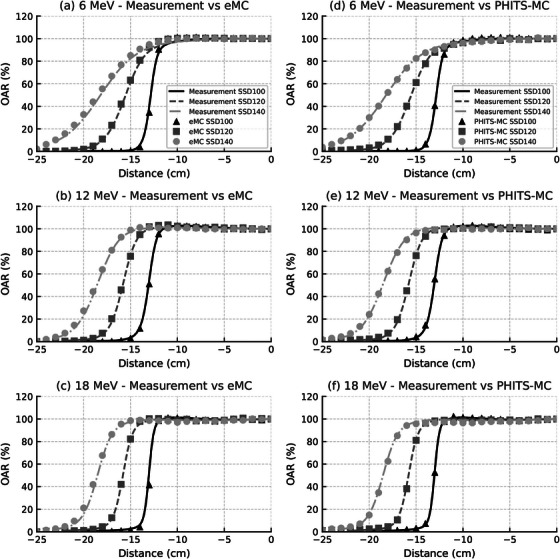
OAR profiles at SSDs of 100, 120, and 140 cm for 6, 12, and 18 MeV electron beams. Panels (a)–(c) compare measured values with eMC simulations, and panels (d)–(f) compare measured values with PHITS‐MC simulations. The OAR values were normalized to 100% at the CAX for each energy. To ensure consistent comparison, all profiles are plotted on the same scale (0%–120%). Black, dark gray, and light gray curves represent SSDs of 100, 120, and 140 cm, respectively. All OAR values were assessed at D_max_.

**FIGURE 7:**
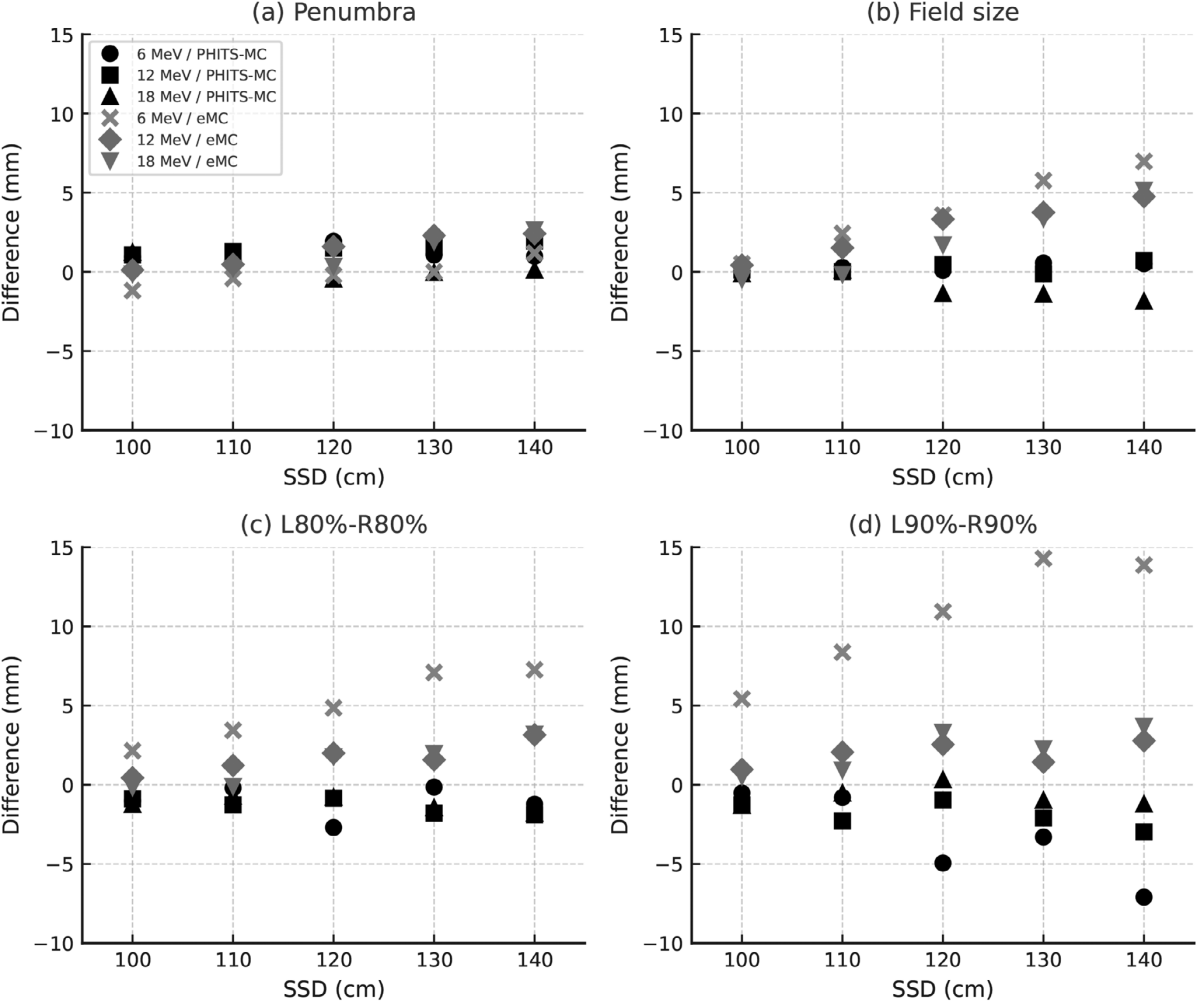
Differences between measured and calculated OAR‐derived parameters as a function of SSD for 6, 12, and 18 MeV electron beams. (a) Penumbra, (b) Field size, (c) L_80%_–R_80%_ distance, and (d) L_90%_–R_90%_ distance. Each symbol shape represents a specific energy and calculation method: circles, squares, and triangles indicate PHITS‐MC results, whereas crosses, diamonds, and inverted triangles represent eMC results. All OAR‐derived parameters were assessed at D_max_. Difference (mm) = Simulation value − Measurement value.

Figure 6 presents OAR profiles for selected SSDs (100, 120, and 140 cm) to improve clarity. Table 4 presents the penumbra, field size, L_80%_–R_80%_ distance, and L_90%_–R_90%_ distance values, with the differences (mm) shown in Figure 7.

**TABLE 4:** Measured and simulated OAR parameters for extended SSDs.

(a) Penumbra (cm)		SSD (cm)					
Energy (MeV)	Penumbra (cm)	100	105	110	120	130	140
6	Measurement	1.2	1.6	2.1	3.2	4.4	5.7
	eMC	1.0	1.5	2.0	3.2	4.4	5.8
	PHITS‐ MC	1.2	1.6	2.1	3.4	4.5	5.8
12	Measurement	1.2	1.4	1.6	2.0	2.6	3.2
	eMC	1.2	1.4	1.6	2.2	2.8	3.5
	PHITS‐MC	1.3	1.5	1.7	2.2	2.8	3.4
18	Measurement	0.8	0.9	1.1	1.6	1.9	2.4
	eMC	0.8	1.0	1.1	1.6	2.1	2.7
	PHITS‐MC	0.9	1.1	1.2	1.5	1.9	2.4

(b) Field size (cm)		SSD (cm)					
Energy (MeV)	Field size (cm)	100	105	110	120	130	140
6	Measurement	25.5	26.8	28.2	30.9	33.5	36.2
	eMC	25.5	27.0	28.4	31.2	34.1	36.9
	PHITS‐MC	25.5	26.9	28.2	30.9	33.5	36.2
12	Measurement	26.0	27.4	28.7	31.4	34.2	36.9
	eMC	26.0	27.4	28.9	31.8	34.6	37.4
	PHITS‐MC	26.0	27.4	28.7	31.5	34.2	37.0
18	Measurement	25.9	27.3	28.6	31.4	34.1	36.9
	eMC	25.8	27.2	28.5	31.6	34.5	37.4
	PHITS‐MC	25.9	27.2	28.6	31.3	34.0	36.7

(c) L_80%_ ― R_80%_ distance (cm)		SSD (cm)					
Energy (MeV)	L_80%_ ― R_80%_ (cm)	100	105	110	120	130	140
6	Measurement	24.3	25.2	26.1	27.6	29.1	30.5
	eMC	24.5	25.5	26.4	28.1	29.8	31.2
	PHITS‐MC	24.2	25.2	26.0	27.4	29.0	30.3
12	Measurement	24.9	26.1	27.2	29.5	31.7	33.8
	eMC	24.9	26.2	27.4	29.7	31.9	34.2
	PHITS‐MC	24.8	26.0	27.1	29.4	31.5	33.6
18	Measurement	25.1	26.3	27.5	29.9	32.2	34.5
	eMC	25.1	26.3	27.5	30.1	32.4	34.8
	PHITS‐MC	25.0	26.2	27.4	29.8	32.1	34.3

(d) L_90%_ ― R_90%_ distance (cm)		SSD (cm)					
Energy (MeV)	L_90%_ ― R_90%_ (cm)	100	105	110	120	130	140
6	Measurement	23.4	24.0	24.5	25.4	26.1	26.9
	eMC	24.0	24.7	25.3	26.5	27.5	28.3
	PHITS‐MC	23.4	23.9	24.4	24.9	25.8	26.2
12	Measurement	24.3	25.4	26.5	28.5	30.4	32.2
	eMC	24.4	25.5	26.7	28.7	30.5	32.5
	PHITS‐MC	24.1	25.2	26.2	28.4	30.2	31.9
18	Measurement	24.6	25.8	26.9	28.9	31.0	33.0
	eMC	24.7	25.9	27.0	29.3	31.3	33.4
	PHITS‐MC	24.5	25.6	26.8	29.0	30.9	32.9

As the SSD is extended, the penumbra increases, with this effect being more noticeable at lower energies. The PHITS‐MC calculations remained accurate within 2 mm of the measured values even when the SSD was extended to 140 cm. However, for eMC, differences greater than 2 mm were observed when the SSD exceeded 130 cm. The field size increased as the SSD was extended to 140 cm, with the 6 MeV field size increasing to 36.2 cm and both 12 MeV and 18 MeV field sizes increasing to 36.9 cm. For PHITS‐MC, the error remained within 2 mm even at an SSD of 140 cm, but for eMC, the error increased with the SSD extension, showing differences of +7, +4.8, and +5.2 mm for 6, 12, and 18 MeV, respectively, at an SSD of 140 cm.

At an SSD of 140 cm, the L_80%_–R_80%_ and L_90%_–R_90%_ distances were 30.5 and 26.9 cm for 6 MeV, 33.8 and 32.2 cm for 12 MeV, and 34.5 and 33.0 cm for 18 MeV. Even in the PHITS‐MC simulation, differences greater than 2 mm were noted for 6 MeV and 12 MeV when the SSD exceeded 120 cm. For eMC, the difference from the measured values was especially large for 6 MeV, with the difference increasing significantly as the SSD was extended. Consequently, at an SSD of 140 cm, the differences in the L_80%_–R_80%_ and L_90%_–R_90%_ distances were +7.3 and +13.9 mm, respectively.

Based on the parameter‐based evaluation, PHITS‐MC satisfied the 3 mm and 3% criteria for all energies even at an SSD of 140 cm. In contrast, eMC failed to meet the 3 mm field size criterion at SSDs ≥ 120 cm across all energies.

### 3.4 OPF for extended SSD

Table 5 shows the measured OPF for the extended SSDs and the differences (%) from the eMC and PHITS‐MC simulations. The OPF values obtained from the eMC and PHITS‐MC simulations were consistent with the measured values within 3%, irrespective of the SSD. However, OPF values from PHITS‐MC simulations tended to be higher than the measured values, whereas those from eMC simulations were generally lower.

**TABLE 5:** Differences (%) between the measured and simulated OPF values for extended SSDs.

		SSD (cm)					
Energy (MeV)	OPF (%)	100	105	110	120	130	140
6	Measurement	100	90.4	81.9	67.9	56.9	48.6
	*eMC difference (%)*		*−0.5*	*−1.0*	*−1.0*	*−1.3*	*−1.4*
	*PHITS‐MC difference (%)*		*0.7*	*1.6*	*2.4*	*1.8*	*1.7*
12	Measurement	100	90.8	82.4	68.9	57.9	49.7
	*eMC difference (%)*		*−0.6*	*−1.4*	*−2.1*	*−0.9*	*−1.7*
	*PHITS‐MC difference (%)*		*0.1*	*0.8*	*1.6*	*2.5*	*1.3*
18	Measurement	100	90.8	82.8	68.6	57.8	49.7
	*eMC difference (%)*		*−0.1*	*−0.3*	*−1.6*	*−0.9*	*−1.6*
	*PHITS‐MC difference (%)*		*0.5*	*0.3*	*1.2*	*1.8*	*1.8*

Difference(%) = Simulation value − measurement value.

## 4 DISCUSSION

The accuracy of dose calculation algorithms is typically assessed by comparing their results with dosimeter measurements or MC simulations. The results obtained support the high accuracy of the eMC algorithm under the standard conditions (SSD = 100 cm), aligning with findings from Hu et al.^20^ who reported agreement within 3 mm between measured PDD and profile values using a silicon diode dosimeter. Furthermore, the commissioning process conducted at our institution's RTPS was validated as appropriate for eMC.

While the 3 mm/3% gamma analysis is a commonly used clinical benchmark to assess dose distribution agreement, it should be noted that this metric may mask localized discrepancies, particularly in steep dose gradient regions. Therefore, in this study, a parameter‐based evaluation following Hu et al.^20^ was employed instead of gamma analysis, to provide a transparent assessment of dose calculation accuracy under homogeneous water phantom conditions. This approach allowed us to identify specific limitations of the eMC algorithm. While PDD and OPF accuracy met the acceptance criteria across all SSDs, and OAR penumbra and mean dose difference within the central 80% of the field were within the defined thresholds, eMC exhibited deviations in field size beyond 3 mm for SSD ≥ 120 cm. These results highlight the importance of careful evaluation when using eMC under extended SSD conditions, especially for large‐field electron beam therapy.

Several studies have assessed the dose calculation accuracy of the eMC algorithm under extended SSD conditions. Fix et al.,^12^ Chamberland et al.,^13^ and Ojala et al. ^14^ reported generally good agreement in PDDs and central‐axis doses under clinical geometries with moderate SSD extensions (110–120 cm), despite discrepancies in low‐energy beams and heterogeneous structures. Further, Ding et al. ^15^ assessed an early version of the eMC algorithm based on a macro MC framework and revealed monitor unit accuracy within 2% up to an SSD of 120 cm; however, their evaluation was limited to central‐axis doses. O'Shea et al. ^16^ employed BEAMnrc to investigate output and scatter variations under extended SSD conditions, but no study has systematically assessed geometric parameters based on OAR profiles. This study is unique because of its systematic validation of eMC dose calculation accuracy for single‐field electron beam irradiation of large PTVs—such as postoperative keloids—requiring SSD extension up to 140 cm.

To evaluate dose distributions in large irradiation fields, the SSD was extended to 140 cm. The eMC‐calculated dose distributions exhibited characteristic changes with SSD extension, including reduced surface dose and an expanded penumbra. The treatable area also increased as the irradiation field expanded. However, at lower energies, penumbra expansion was more pronounced, indicating that treatment field size does not necessarily scale linearly with irradiation field size. Notably, the reduced surface dose observed in the simulations, particularly in the buildup region, may be partly attributed to differences in dose assessment methods. The measurements were obtained using a finite‐sized dosimeter, which is subject to volume‐averaging and perturbation effects, whereas the eMC and PHITS‐MC simulations provide point dose calculations without such effects. These methodological differences may contribute to surface dose discrepancies between measurement and simulation.

Detailed reports on PDD and OAR at extended SSDs remain scarce. This study contributes valuable insights in this context. PDD values calculated by eMC agreed with measurements within approximately 2 mm at an SSD of 140 cm, demonstrating high accuracy along the central axis. This accuracy may stem from the use of a large applicator, ensuring LSE. However, OAR calculations showed reduced accuracy in peripheral regions compared with PHITS‐MC simulations, particularly at low energies (6 MeV), where discrepancies became more evident with SSD extension.

These trends are consistent with previous reports. Aubry et al. noted that eMC showed poorer agreement with film measurements compared to EGSnrc, especially at 6 MeV. ^7^ Ding et al. also pointed out that the eMC beam model insufficiently accounts for scattered electrons from applicator edges, reducing accuracy in the out‐of‐field dose region at low energies.^15^　Large irradiation fields are known to influence beam characteristics. Tzedakis et al.,^27^ using MC simulations of 6 MV x‐rays, demonstrated that in large irradiation fields, the mean energy of incident electrons and radial intensity distribution impact the shape of profile edges. Similarly, in electron beams, beam modeling parameters can contribute to uncertainties. O'Shea et al.^16^ analyzed extended SSDs using the DOSXYZnrc code and found that large fields exhibited increased contributions from steeply scattered electrons. For electron beams, extended SSDs significantly affect the amount and direction of scattered electrons, depending on field size and energy. These factors may amplify uncertainties related to applicator modeling and scattered radiation. In this study, SSD expansion further reduced eMC calculation accuracy.

With regard to the evaluation of OPF, both eMC and PHITS‐MC calculations agreed with measurements within 3% even at SSD of 140 cm, thereby meeting the criteria set forth by the AAPM Medical Physics Practice Guideline 5.a.^26^ Notably, the use of absolute dose measurements at D_max_ in the eMC algorithm for source intensity weighting may have contributed to stable OPF prediction even at extended SSDs. While all PDD and OAR profiles in this study were normalized to D_max_ for visual clarity and consistency, we acknowledge that this normalization may mask systematic offsets in absolute dose. However, absolute dose calibration was separately performed during the commissioning process using measured D_max_ values under reference conditions. Therefore, potential systematic offsets in the eMC output were independently addressed and accounted for in MU calibration. This allowed the geometric evaluation in this study to focus on relative depth and shape characteristics. Although not intended to represent clinical dose delivery, this approach supports commissioning‐level assessment of algorithm behavior. A more detailed investigation of normalization effects in the context of absolute dose accuracy may be warranted in future studies.

Overall, these results demonstrate that the eMC algorithm provides accurate dose calculations along the central axis and for output factors under extended SSD conditions in homogeneous water phantoms. However, reduced accuracy was observed in the peripheral regions of OAR profiles at lower energies and with increasing SSD. These findings clarify the strengths and limitations of the eMC algorithm under these specific conditions and provide useful information for algorithm evaluation and quality assurance in large‐field electron beam therapy planning.

This study was conducted using homogeneous, water‐equivalent phantoms, and future work should extend assessments to heterogeneous anatomical structures and patient‐specific cases. Particularly, efforts should be directed toward improving calculation accuracy in low‐energy beams and at field edges, as well as enhancing computational efficiency under extended SSD settings.

## 5 CONCLUSION

In this study, the accuracy of the eMC dose calculation algorithm was systematically evaluated under extended SSD conditions using homogeneous water‐equivalent phantoms. The results showed that eMC maintained high accuracy for PDD and OPF across all SSDs up to 140 cm. However, notable deviations in lateral dose profiles were observed at SSDs ≥ 120 cm, particularly for low‐energy beams such as 6 MeV.

These findings clarify the performance characteristics and limitations of the eMC algorithm under extended SSD conditions in large‐field electron beam therapy.While the evaluation was conducted in homogeneous phantoms, the parameter‐based assessment provides a transparent framework for algorithm quality assurance. The results provide valuable guidance for algorithm selection and quality assurance practices, and serve as a foundation for future studies involving clinical cases and heterogeneous geometries to further validate the clinical robustness of the eMC algorithm.

## AUTHOR CONTRIBUTIONS

Naohito Ono: Led the conceptualization of the study, performed data collection and analysis, and prepared the manuscript. Makoto Omiya: Conducted modeling using PHITS for MC simulations and validated the model through comparison with experimental data. Manami Sugiyama: Measured radiation dose data and validated the eMC simulation results using the collected data. Masaki Oshima: Supervised the study and critically reviewed the final manuscript.

## CONFLICT OF INTEREST STATEMENT

The first author and all co‐authors declare no conflicts of interest.

## Data Availability

The data that support the findings of this study are available from the corresponding author upon reasonable request.
